# A phase I/II study of siltuximab (CNTO 328), an anti-interleukin-6 monoclonal antibody, in metastatic renal cell cancer

**DOI:** 10.1038/sj.bjc.6605872

**Published:** 2010-08-31

**Authors:** J-F Rossi, S Négrier, N D James, I Kocak, R Hawkins, H Davis, U Prabhakar, X Qin, P Mulders, B Berns

**Affiliations:** 1 Service d'Hématologie et d'Oncologie Médicale, CHU Saint-Eloi, BT 509-INSERM U847, CHRU Montpellier et Université Montpelllier I, Montpellier, France; 2 Département d'Oncologie Médicale, Centre Léon-Bérard, University of Lyon, CIC 28, rue Laënnec- 69373, Lyon Cedex 08, France; 3 Clinical Oncology, Queen Elizabeth Medical Centre, Edgbaston, Birmingham B15 2TH, UK; 4 Clinic of Complex Oncological Care, Masaryk Memorial Cancer Institute, Žluý kopec Street 7, Brno, 656 53, Czech Republic; 5 Medical Oncology, Christie Hospital and Medical Oncology, University of Manchester, Christie Research Centre, Wilmslow Road, Manchester M20 4BX, UK; 6 Clinical Pharmacology, Biostatistics, Centocor, Research and Development, Inc., 105 Great Valley Corporate Center, Malvern, PA 19355, USA; 7 Department of Urology, Radboud University Medical Center, PO Box 9101, Nijmegen 6500HB, The Netherlands; 8 Oncology, Centocor Research and Development, 50-100 Holmers Farm Way, High Wycombe, Buckinghamshire HP12 4DP, UK

**Keywords:** metastatic renal cell cancer, interleukin-6, siltuximab, C-reactive protein

## Abstract

**Background:**

Serum interleukin (IL)-6 levels correlate with disease outcomes in renal cell carcinoma (RCC) patients. Siltuximab, a chimeric, murine-human mAb against IL-6, was evaluated in a three-part phase I/II study in patients with progressive metastatic RCC.

**Methods:**

In part 1, 11 patients received 1, 3, 6, or 12 mg kg^–1^ at weeks 1, 4 and q2w × 2 thereafter; in part 2, 37 patients randomly received 3 or 6 mg kg^–1^ q3w × 4; in part 3, 20 low-risk patients received 6 mg kg^–1^ q2w × 6. Modified WHO response criteria were assessed at weeks 7, 11, the 6-week follow-up, and when clinically indicated.

**Results:**

Siltuximab was well tolerated overall, with no maximum tolerated dose or immune response observed. In all, 5 out of 11, 17 out of 37, and 9 out of 20 patients in parts 1, 2, and 3, respectively, received extended treatment beyond 4–6 initial infusions. In part 2, stable disease (SD) (⩾11weeks) or better was achieved by 11 out of 17 (65%) 3 mg kg^–1^ treated patients (one partial response (PR) ∼8 months, 10 SD) and 10 out of 20 (50%) 6 mg kg^–1^ treated patients (10 SD). In part 3, documented complete or PR was not observed, but 13 out of 20 (65%) patients achieved SD.

**Conclusion:**

Siltuximab stabilised disease in >50% of progressive metastatic RCC patients. One PR was observed. Given the favourable safety profile of siltuximab and poor correlation of tumour shrinkage with clinical benefit demonstrated for other non-cytotoxic therapies, further evaluation of dose-escalation strategies and/or combination therapy may be considered for patients with RCC.

The inflammatory cytokine interleukin (IL)-6, is part of an autocrine cytokine network that influences tumour growth. The production of cancer-related factors supporting IL-6 production by the tumour correlate with its propensity to establish distant metastases. In particular, metastatic renal cell carcinoma (RCC) is frequently associated with elevated levels of IL-6, which have been reported to correlate with metastatic progression, poor prognosis, shorter survival (Blay *et al*, 1992; Negrier *et al*, 2004), as well as poor response (Fumagalli *et al*, 1999) and high toxicity (Capuron *et al*, 2001) to IL-2 therapy.

An estimated 30–40% of RCC cases eventually advance to metastatic disease (Glaspy, 2002). This is often accompanied by paraneoplastic syndrome, a condition characterised by fever, elevated levels of acute phase markers such as C-reactive protein (CRP), a decrease in serum albumin, anaemia, and thrombocytosis, all of which appear to be because of abnormal cytokine production, in particular IL-6, or immunogenic mechanisms (Blay *et al*, 1997; Altundag *et al*, 2005). Until recently, cytokines (IL-2 and interferon-*α* (IFN*α*)) were the only drugs shown to induce tumour regression in patients with metastatic RCC. Today, prolongation of the time to progression in patients with RCC has been demonstrated for the tyrosine kinase inhibitors sorafenib (Escudier *et al*, 2007a), sunitinib (Motzer *et al*, 2007), as well as temsirolimus (Hudes *et al*, 2007) and bevacizumab (Escudier *et al*, 2007b). However, these agents are associated with significant adverse effects, and the need for curative therapy remains.

Monoclonal antibodies against IL-6 have been studied for more than one decade. BE-8 (Diaclone, Besançon, France), a fully murine monoclonal antibody against IL-6, inhibited RCC- and IL-2/IFN*α*-associated toxicities in preliminary reports of testing in patients with metastatic RCC (Rossi *et al*, 1992; Blay *et al*, 1997). However, human anti-murine antibodies to BE-8 were detected (Legouffe *et al*, 1994), and daily production of IL-6 levels >18 *μ*g day^–1^ could not be efficiently blocked with the doses of BE-8 tested (Lu *et al*, 1995). Thus, further exploration of chimeric or fully human anti-IL-6 antibodies was warranted.

Siltuximab (formerly known as CNTO 328) is a chimeric, murine-human monoclonal antibody that binds with high affinity and specificity to IL-6 (van Zaanen *et al*, 1996). Preclinical experience has shown that siltuximab inhibits the growth of human RCC tumours and decreases serum calcium concentrations in nude mice (Weissglas *et al*, 1995). An earlier formulation of siltuximab was evaluated in a phase I, dose-ranging study of 10, 20, or 40 mg daily infusions administered in two 2-week cycles to 12 patients with multiple myeloma. This study demonstrated biological activity (van Zaanen *et al*, 1998). Eight of nine patients with detectable CRP concentrations showed a reduction to a level below detection, and all nine patients showed suppression of circulating free IL-6 60 days post-treatment. The antibody was well tolerated, and no immune responses to siltuximab were observed. These results were encouraging and supported further evaluation of the antibody in less frequent and longer-term dosing.

In this paper, we present the clinical data from the first-in-human, phase I/II study of this formulation of siltuximab in continuous dosing, conducted in patients with metastatic RCC. The objectives of this study were to evaluate the safety, pharmacokinetics, and preliminary efficacy of siltuximab in the treatment of patients with metastatic RCC and to determine the recommended dose for further studies. In-depth pharmacokinetic and pharmacodynamic results of the study have been published separately (Puchalski *et al*, 2010).

## Materials and methods

### Study design

This study was conducted in three parts. Part 1 was a phase I, open-label dose-escalation study to assess safety and single-dose pharmacokinetics of siltuximab at three dose levels (1, 3, and 6 mg kg^–1^ on days 1, 29, 43, and 57) and to determine the two highest safe doses for further evaluation in part 2. The starting dosage regimen for part 1 of this phase I/II study was selected in an attempt to sufficiently neutralise the circulating level and endogenous production of IL-6. Maximum suppression of CRP concentrations to below the lower limit of quantification (LLOQ) throughout the dosing interval was anticipated to provide the best opportunity for CNTO 328 therapy to demonstrate clinical activity (Puchalski *et al*, 2010).

Enrolment was initiated with the lowest dose cohort and only proceeded to the next dose level after the Safety Monitoring Committee (SMC) reviewed safety data and recommended dose escalation.

On recommendation of the SMC, the protocol was later amended to explore a fourth dose, 12 mg kg^–1^, in patients with high (⩾50 mg l^–1^) baseline serum CRP at one centre only. The enrolment of this cohort coincided with the enrolment for part 2 and was not considered for dose selection.

Part 2 was a proof-of-concept, phase II, randomised, double-blind, Simon 2-stage design to evaluate two safe siltuximab dose levels from part 1 (3 and 6 mg kg^–1^ administered 1 : 1 q3w for four cycles). As both treatment cohorts met the effective dose criterion in stage 1, the independent Safety and Efficacy Monitoring Committee decided not to proceed to stage 2 of part 1. An open-label part 3 was added to further evaluate 6 mg kg^–1^ siltuximab at a more frequent dosage regimen (q2w for six cycles) that would most likely completely neutralise circulating free IL-6 (as indicated by CRP suppression) in a more select patient population (i.e., low risk with Motzer score ⩽1 and CRP levels <30 mg l^–1^).

For all study parts, patients demonstrating at least stable disease (SD) could receive extended administrations of siltuximab at the assigned dose (q3w for ⩽4 total infusions (part 1), or q3w (part 2) or q2w (part 3) for up to ⩽6 infusions). The study agent was stopped for any patient with disease progression. After the last siltuximab infusion, patients were followed for 24 weeks for pharmacokinetics, pharmacodynamics, and immune response evaluations; and every 2 months for up to 1 year for disease status, post-study treatments, and survival.

### Patient population

Patient eligibility criteria were: age ⩾18 years, clinically diagnosed metastatic RCC with documented metastases beyond the regional lymphatics (any T, any N, M1 disease); detectable CRP serum concentrations ⩾4 mg l^–1^ (and <30 mg l^–1^ for part 3); measurable disease (and/or evaluable disease for part 1); documented disease progression based on objective tumour assessments from two computed tomography scans within 6 months before enrolment; Karnofsky performance status ⩾60; Motzer score (Motzer *et al*, 2004) ⩽1 (part 3 only); ⩾4 weeks wash-out since previous systemic cancer therapy, radiotherapy, or surgery; adequate bone marrow, liver, and renal function (haemoglobin ⩾9 g per 100 ml, white blood cells ⩾3.5 × 10^9^ l^–1^, neutrophils ⩾1.5 × 10^9^ l^–1^, platelets ⩾100 × 10^9^ l^–1^, serum creatinine ⩽2 mg per 100 ml, liver function tests ⩽2 × upper limit of normal (ULN) if no liver metastasis or ⩽5 × ULN if liver metastasis); active birth control for women of child-bearing potential; and life expectancy ⩾6 months.

Exclusion criteria were the use of any investigational drug within 30 days or five half-lives; any history of receiving murine/chimeric proteins or human/murine recombination products; serious concurrent illness or significant cardiac disease characterised by significant ischaemic coronary disease or congestive heart failure; chronic, recurrent, or clinically important active infection; solid organ transplant (except corneal >3 months before screening), allogeneic bone marrow transplant, or peripheral blood stem cell transplant; central nervous system metastatic disease; previous non-RCC malignancy except adequately treated basal/squamous cell skin carcinoma, cervical cancer, or other cancer in remission for ⩾5 years; other concurrent immunotherapy, biotherapy, chemotherapy, investigative therapy, radiotherapy, or immunosuppressive therapy; planned concomitant nephrectomy; pregnant or lactating; known HIV, hepatitis B or C; autoimmune disease; or any medical condition that in the investigator's opinion might compromise patient adherence to the study agent.

The protocol was approved by the independent ethics committee at each site. All patients provided written informed consent.

### Study assessments

#### Safety

Primary safety assessments included the monitoring of siltuximab-related dose-limiting toxicity (DLT) according to the National Cancer Institute Common Toxicity Criteria Version 2.0, Bethesda, MD, USA; adverse events (AEs); serious adverse events (SAEs); and clinically significant changes from baseline in vital signs, safety-related laboratory parameters, and abnormal electrocardiograms.

In part 1, a 48-h safety assessment was performed after the first administration to the first patient in each cohort before additional patients could be enrolled. If no DLT (any grade ⩾3 toxicity identified by the SMC as dose limiting) was observed, the doses were considered safe and no maximum tolerated dose (MTD) was reached. If a DLT was observed at a lower dose level after escalation, the SMC would make a recommendation on which dose level, if any, to continue to study.

#### Pharmacology

Blood samples to determine pharmacokinetics and pharmacodynamics of CNTO 328 were obtained from all patients within 2 weeks before the first infusion and during and in between each infusion visit for analysis. Although CRP is not a validated biomarker in RCC patients, we believe CRP suppression is a meaningful surrogate marker for demonstrating inhibition of IL-6 signaling. Serum amyloid A (SAA), also induced by IL-6 as an acute-phase inflammatory protein, and soluble IL-6 receptor (GP80, sIL-6R) and GP130 were investigated as other potential pharmacodynamic biomarkers of anti-IL-6 therapy, because these biomarkers are possibly more sensitive than CRP to IL-6 changes. Increased GP130 serum concentrations are associated with increased IL-6 in rheumatoid arthritis (Tanaka *et al*, 2000). As IL-6 affects bone resorption, which is generally associated with increased circulating serum C-telopeptide (CTx) and N-telopeptide (NTx) levels of collagen, additional pharmacodynamic data on serum NTx and CTx were measured by ELISA.

Detailed results on siltuximab pharmacokinetics and the serum concentrations of the pharmacodynamic markers CRP, SAA, and IL-6 are presented separately in the pharmacokinetic/pharmacodynamic modeling paper, because the assessment of CRP suppression was used to determine the optimal dose levels of siltuximab and SAA was positively correlated with CRP and therefore was considered a good alternative biomarker of siltuximab treatment (Puchalski *et al*, 2010). Data on soluble GP130, NTx, and CTx are presented here.

Additional blood samples were collected from all patients immediately before the first infusion and at 6, 12, 18, and 24 weeks after the last infusion to evaluate the presence of antibodies to siltuximab, that is, any increase from baseline in anti-siltuximab antibody titre in which the increased reactivity demonstrated specific binding to siltuximab.

#### Efficacy

Overall tumour response was assessed using modified World Health Organisation (WHO) criteria through pertinent radiologic examination (excluding ultrasound) of bidimensionally measurable lesions. Complete response (CR) and partial response (PR) required confirmation by repeat radiologic assessment ⩾4 weeks later. In part 1, the assessments were performed at baseline, week 9, and at the 6-week follow-up. For parts 2 and 3, radiologic assessments were performed at baseline, week 7, week 11 (to confirm a potential response), when clinically indicated, and 6 weeks after the last siltuximab administration. The same radiological method was to be used during all timepoints.

The primary efficacy endpoint was the proportion of patients with a best response of CR, PR, or SD for parts 1 and 2 and of CR or PR for part 3, both within 11 weeks after the first siltuximab administration. A more stringent endpoint was chosen for part 3 because better response was expected in the highly selected patients.

Major secondary endpoints included the time to progressive disease (PD), the proportion of patients with CR or PR, the duration of CR or PR, and the proportion of patients with clinical benefit. For this study, clinical benefit response was defined as improvement in ⩾1 and stable condition for the remaining criteria as follows: ⩾2-unit improvement in worst pain measurement according to question 3 of the Brief Pain Inventory short form, ⩾10% absolute improvement from baseline in Karnofsky performance status, ⩾10% increase from baseline in body weight. This definition of clinical benefit response has not been formally validated.

The functional assessment of chronic illness therapy – fatigue questionnaire was also administered.

### Statistical analyses

Summary statistics were provided for continuous data, and rates and percentages for categorical data. Kaplan–Meier survival analyses were performed for time-to-event data. Baseline was defined as the last measurement before the first siltuximab administration. Formal hypothesis testing was performed only for the primary efficacy endpoints in parts 2 and 3. All statistical analyses were performed by BioCor using SAS Version 8.2 (SAS Institute Inc., Cary, NC, USA).

For part 1, a sample size of 3–21 patients was planned to allow for cohort expansion in the event of a DLT. For part 2, according to optimal Simon 2-stage design, the planned sample size of ⩾18 patients per group for stage 1 and an additional 25 patients for stage 2 was calculated based on the null hypothesis that <10% of patients would achieve response (CR, PR, unconfirmed CR or PR, or SD) and the alternative hypothesis that ⩾25% would achieve response at an *α*-level of 0.05 and *β*-level of 0.20. Under these assumptions, the study would proceed to stage 2 if >2 patients in a dose cohort achieved response. A dose was effective if >7 patients overall (stages 1 and 2) in that dose cohort achieved response. For part 3, the planned sample size of 20 patients was calculated based on the null hypothesis that <5% of patients would achieve response (CR or PR) and the alternative hypothesis that ⩾25% would achieve response at an *α*-level of 0.10 and *β*-level of 0.10. Under these assumptions, siltuximab would be considered effective if ⩾3 patients achieved response in part 3.

## Results

A total of 68 patients were enrolled from June 2003 to May 2006 across parts 1 through 3 at multiple European sites. Baseline demographics and disease characteristics were well balanced across the study parts (Table 1).

Eleven patients were enrolled in part 1: one patient received 1 mg kg^–1^, three patients each received 3 or 6 mg kg^–1^, and four patients received 12 mg kg^–1^. In all, 9 of these 11 patients had unilateral nephrectomy for RCC, and 4 had received radiotherapy for metastatic disease. Seven patients had been treated with IFN*α* and four with the combination of IFN*α* and IL-2. The highest number of previous cancer-related systemic therapies used was three (in two patients), and the best response to the last systemic therapy was SD (*n*=4). Of these 11 patients, 8 received all 4 planned study-agent infusions, including 5 who went on to receive extended treatment (4 received 4 extended infusions and 1 received 1 extended infusion). The median time from the first to the final study-agent administration for the treatment cohorts combined was 57 days (range 1–64 days).

**Figure 1:**
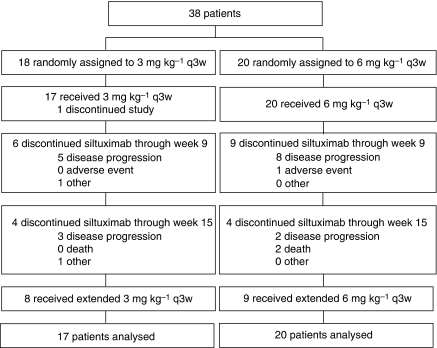
CONSORT diagram for the randomised part 2.

A total of 38 patients were randomised in part 2: 18 patients to 3 mg kg^–1^ and 20 patients to 6 mg kg^–1^, although one patient assigned to 3 mg kg^–1^ did not meet entry criteria and consequently did not receive any study agent (Figure 1). All but three patients had previous surgery for RCC. In all, 30 patients had previous cancer-related systemic therapy, 22 of whom had ⩽2 regimens. Among patients who had >2 regimens, six patients had received three regimens, one patient had received four regimens, and one patient in the 3 mg kg^–1^ dose group had received nine regimens. The most commonly used systemic therapy was cytokine-based (e.g., IFN*α*, IL-2), whereas fewer patients had received chemotherapy or hormonal therapies; the best response achieved was PR for four patients in the 3 mg kg^–1^ group. More patients had previous radiation therapy in the 6 mg kg^–1^ group than in the 3 mg kg^–1^ group (8 *vs* 1, respectively). Among the 37 treated patients, 23 received all four planned infusions, including 17 who went on to receive at least one extended treatment (one patient with PR received the maximum of six extended treatments over 18 weeks). The median time from the first to the final administration for the treatment groups combined was 71 days (range 22–233 days).

In part 3, 20 patients (at baseline 12 had a Motzer score of 0 and 8 had a score of 1) received 6 mg kg^–1^ siltuximab. All patients had unilateral nephrectomy, and one had received radiotherapy. In total, 18 patients had received cancer-related systemic therapy; 16 patients had been treated with IFN*α*, among whom 10 had received a combination of IFN*α* and an IL. Only one patient received >2 regimens. The best responses achieved with the last systemic therapy were CR (*n*=1) and PR (*n*=3). Among these 20 patients, 13 received all 6 planned infusions, including 9 who went on to receive at least one extended treatment (one patient received 7 extended treatments over 14 weeks). The median time from the first to the final administration was 71 days (range 1–239 days).

### Safety

Siltuximab was well tolerated and demonstrated a consistent safety profile across all cohorts and each study part. Administration of 6 mg kg^–1^ q3w in part 2 and q2w in part 3 did not result in significantly different safety profiles. No DLT, dose-related toxicity, or dose response for any specific AE or system-class AE was observed. No MTD was established.

Almost all treated patients (66 of 68) in parts 1 through 3 had ⩾1 AE (Table 2). The majority of individual AEs were not reported by more than one patient each. Reasonably related AEs were reported in 46 patients; fatigue (*n*=9) and dizziness (*n*=5) were most frequently reported. Twenty-seven (40%) patients had ⩾1 AE of toxicity grade ⩾3. In part 1, all grade ⩾3 AEs were reported by only one patient each, and none were considered likely related to the study agent. In part 2, the majority of grade ⩾3 AEs were reported by only one patient per treatment group; the most frequently reported were fatigue, chest pain, back pain, dyspnoea, and hypertension (each *n*=2). In part 3, four patients had one or more grade ⩾3 AEs (transaminases increased, syncope, pain, musculoskeletal pain, and hypercalcaemia (each *n*=1)). Of the reasonably related AEs reported, eight were grade ⩾3 in part 2 (pharyngitis and bronchitis (each *n*=2); hypertension, fatigue, cough, and upper abdominal pain (each *n*=1)) and two were grade ⩾3 in part 3 (pain and syncope (each *n*=1)).

Serious AEs were reported in 5 (46%) patients in part 1, 12 (32%) in part 2, and 3 (15%) in part 3. One patient in part 2 who received three infusions of 6 mg kg^–1^ siltuximab suffered a grade 4 SAE of cardiac failure on day 78 and consequently withdrew from study participation. This was the only SAE considered possibly related to siltuximab, although the investigator reported inflow obstruction by the large tumour masses in the mediastinum as the most likely cause of cardiac failure. A total of five patients died during the study: four patients died from disease progression: two 12 mg kg^–1^ treated patients in part 1 (on days 31 and 106) and two 6 mg kg^–1^ treated patients in part 2 (on days 45 and 66). One 6 mg kg^–1^ treated patient died from a grade 4 SAE of massive cerebral haemorrhage secondary to metastatic disease in part 3 (on day 107).

No anaphylactic or delayed hypersensitivity reaction was observed. Among the 10 patients who reported possible infusion reactions (Table 2), none required an interruption of the infusion or a discontinuation of subsequent infusions. No treatment-related changes in vital signs (except for increased blood pressure in part 2), electrocardiogram, urinalysis, or clinical chemistry parameters were evident. In parts 2 and 3, trends toward decreases from baseline in the haematology parameters platelets, white blood cells, and neutrophils were observed with prolonged study-agent administration; all three parameters trended toward recovery upon study-agent discontinuation.

#### Pharmacology

Using a stepwise design, pharmacokinetic/pharmacodynamic modeling was used in which a target trough siltuximab serum concentration of 21 *μ*g ml^–1^ was predicted to be effective in maintaining complete CRP suppression throughout the dosing interval (Puchalski *et al*, 2010). In parts 1 and 2, although a decrease from baseline in CRP was evident in both the 3 and 6 mg kg^–1^ cohorts, CRP was inadequately suppressed in both cohorts when a patient's baseline serum CRP was >30 mg l^–1^. Total CRP suppression was also not achieved in the 12 mg kg^–1^ dose cohort in which patients had the highest baseline CRP concentrations (CRP >50 mg l^–1^). The pharmacokinetic/pharmacodynamic modeling simulation showed that dosing at 6 mg kg^–1^ q2w or 9 mg kg^–1^ q3w would suppress CRP to below the LLOQ throughout siltuximab treatment (Puchalski *et al*, 2010). In part 3, administration of siltuximab 6 mg kg^–1^ q2w appeared to suppress CRP in all 20 patients with baseline CRP serum concentrations <30 mg l^–1^.

Briefly summarised from Puchalski *et al*, (2010), SAA concentrations were variable in part 1 and remained low throughout the treatment period in parts 2 and 3; total and free IL-6 serum concentrations gradually increased in parts 1 and 2, and was not measured in part 3. Levels of soluble IL-6R (GP80) and soluble GP130 both increased from baseline for the 3 and 6 mg kg^–1^ cohorts following siltuximab administration in parts 2 and 3. The increase in soluble IL-6R was greater in the 6-mg kg^–1^ than in the 3 mg kg^–1^ dose cohort in part 2. In part 2, patients in the 6 mg kg^–1^ dose cohort generally had higher levels of serum NTx and CTx than those in the 3 mg kg^–1^ dose cohort, and both serum NTx and CTx varied considerably without showing any specific trend. In part 3, an increase in serum levels of both NTx and CTx was evident following the initial siltuximab administrations; both decreased to below baseline levels by the end of the study.

Among the 44 of 68 patients in parts 1 through 3 with one or more samples obtained after the first siltuximab administration, none were positive for antibodies against siltuximab at any time.

### Efficacy

**Figure 2:**
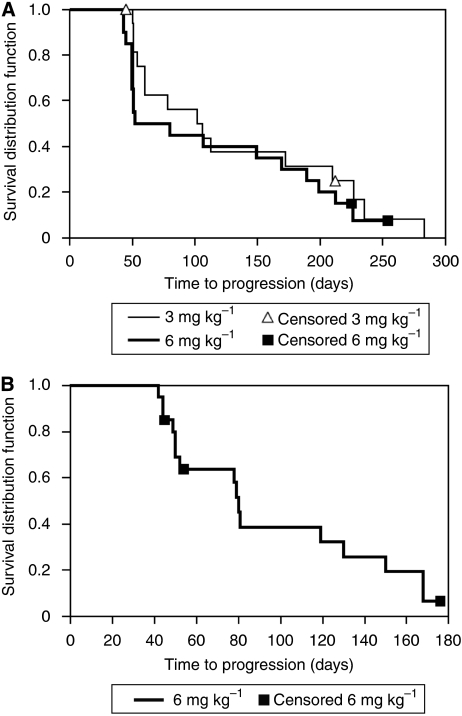
Kaplan–Meier plot of the time to disease progression through the end of study for treated patients in (**A**) part 2 and (**B**) part 3.

In part 1, five patients had an overall response of SD (3 mg kg^–1^, *n*=1; 6 mg kg^–1^, *n*=3; 12 mg kg^–1^, *n*=1). In part 2, the primary endpoint of CR, PR, or SD was achieved: one patient had PR and 20 (10 each in the 3 and 6 mg kg^–1^ cohorts) had SD (Table 3), that is, both cohorts met the effective dose criterion in stage 1. The patient with PR had neck and pancreatic metastases that had progressed despite treatment with high-dose IL-2, IFN*α*, and 5-fluorouracil 2 months before receiving 3 mg kg^–1^ siltuximab. Partial response was observed in the target neck lesion during the first radiologic assessment at week 7 and was sustained for 228 days before radiologic examination on day 283 showed PD. This patient received 10 total infusions (4 scheduled plus 6 extended). Her CRP serum concentration decreased from a baseline concentration of 36 mg l^–1^ to below 10 mg l^–1^ and remained suppressed throughout her treatment. Overall, the median time to PD for all treated patients was 102 (95% CI: 52, 169) days. The time to PD for the patients with SD ranged from 78 to 254 days (Figure 2A). A higher proportion of patients who responded to treatment with siltuximab had low baseline CRP (<30 mg l^–1^) compared with those who had high baseline CRP (46 *vs* 11%). For patients with a CRP serum concentration below the LLOQ at the third infusion, 14 (74%) of 19 had a best response of SD. No patient with a baseline CRP ⩾100 mg l^–1^ had a response of SD or better.

In part 3, the primary endpoint of CR or PR was not achieved, although 13 of 20 patients demonstrated SD (Table 3). The median time to PD for all treated patients was 80 (95% CI: 50, 130) days. For the 13 patients with SD, the time to PD ranged from 78 to 176 days (Figure 2B). Of these patients, four were right-censored because they had no reported PD during the study.

**Figure 3:**
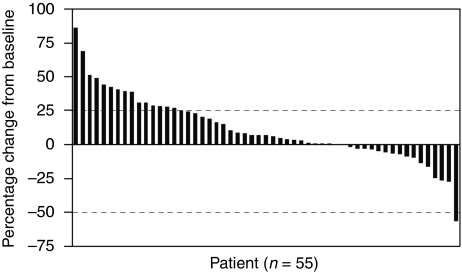
Maximal percentage of tumour reduction according to modified WHO criteria for patients in parts 2 and 3. Dotted lines at 25% and −25% represent the criteria for PD and PR, respectively. Two patients in the 3 mg kg^–1^ group in part 2 were not evaluable because neither had a week 11 radiologic assessment.

The maximum percentage of tumour reduction from baseline is shown for parts 2 and 3 combined in Figure 3. There was no significant difference in tumour reduction from baseline between patients treated with siltuximab administered at 3 mg kg^–1^ q2w, 6 mg kg^–1^ q2w, and 6 mg kg^–1^ q3w.

In part 1, 5 of 10 patients at week 4 (1 mg kg^–1^, *n*=1; 3 mg kg^–1^, *n*=1, 6 mg kg^–1^, *n*=1; 12 mg kg^–1^, *n*=2), 3 of 9 at week 6 (6 mg kg^–1^, *n*=1; 12 mg kg^–1^, *n*=2), and 2 of 8 at week 8 (6 mg kg^–1^, *n*=1, 12 mg kg^–1^, *n*=1) were clinical benefit responders. An early small improvement (∼20%) in clinical benefit response was observed in both parts 2 and 3, although this was difficult to evaluate because of a lack of adequate data beyond 6 weeks. An improvement in fatigue was seen for ∼35% of patients up to week 6 in part 2; this benefit was not seen in part 3, likely because of the low level of fatigue in this population at baseline.

## Discussion

Patients with metastatic RCC may have highly variable presentation and clinical course, however, our study population had advanced, refractory, PD. Almost all treated patients had previous surgery for RCC, the majority had at least one previous systemic cancer treatment, and all had documented PD before study entry. In parts 1 and 2, patients’ baseline clinical features and signs and symptoms profiles were typical of a population with advanced stage RCC (Atzpodien *et al*, 2003; Kim *et al*, 2003; Schips *et al*, 2003; Cella *et al*, 2006). Additionally for part 2, retrospective analysis estimated that one-third of patients had a baseline Motzer score (Motzer *et al*, 2004) of 2. For part 3, it was hypothesised that by selecting low-risk patients (i.e., based on a baseline Motzer score of ⩽1), this sample population would have a greater probability of benefiting from an anti-IL-6 antibody. Thus, in part 3, a low-risk population was enrolled (60% of patients had a Motzer score of 0, 40% of patients had a Motzer score of 1). Additionally, all patients in part 3 had a CRP serum concentration of <30 mg l^–1^.

The majority of patients across all parts of the study had temporary disease stabilisation with siltuximab and one patient in the 3 mg kg^–1^ group of part 2 had a significant tumour response with a PR. In part 2, the primary endpoint (documented CR, PR, or SD within 11 weeks after the first siltuximab administration) was met. Across both parts 2 and 3 of the study, 29% of all evaluable patients demonstrated some degree of reduction in measurable disease during the study period. The median time to PD for all treated patients was 102 (95% CI: 52, 169) days in part 2 and 80 (95% CI: 50, 130) days in part 3. There was a small clinical benefit response in both parts 2 and 3. A benefit in fatigue was only observed in part 2, possibly because of the lower fatigue at baseline observed in the part 3 population. The hypothesis that low-risk patients have a greater probability of benefit from siltuximab was not confirmed in part 3.

Interleukin-6 is a biological mediator of CRP production (Mahmoud and Rivera, 2002). High serum CRP concentrations have been correlated with elevated IL-6 serum concentrations (Pelliniemi *et al*, 1995; Ljungberg *et al*, 1997) and with poor prognosis in solid tumours such as colorectal cancer (Chung and Chang, 2003) and with mortality in RCC (Karakiewicz *et al*, 2007). In addition, CRP has a biological role in IL-6 production and chemoresistance in multiple myeloma (Yang *et al*, 2007).

On the basis of evidence that CRP can be a biomarker for IL-6 (Pelliniemi *et al*, 1995; Ljungberg *et al*, 1997), we hypothesised that suppression of CRP serum concentration to below the LLOQ of 4 mg l^–1^ throughout the dosing interval would demonstrate siltuximab's clinical activity (Puchalski *et al*, 2010). In patients with baseline CRP serum concentrations ⩾30 mg l^–1^, serum CRP was inadequately suppressed (or maintained) with siltuximab 3 and 6 mg kg^–1^ q3w in part 2. Also, a greater percentage of part 2 patients with baseline CRP <30 mg l^–1^ than with baseline CRP ⩾30 mg l^–1^ (46 *vs* 11%) responded to siltuximab treatment. Thus, an important question that arose during this study was whether patients with higher serum CRP (i.e., ⩾30 mg l^–1^) had been given an insufficient siltuximab dose to suppress their IL-6 levels adequately to achieve a tumour response.

Interleukin-6 could not be assessed reliably. Other potential biomarkers of IL-6 inhibition were also examined. Changes in SAA concentrations were positively correlated with CRP (Puchalski *et al*, 2010). The observed dose-dependent increase in GP130 following treatment with siltuximab seemed inconsistent with a decrease in IL-6 bioactivity as measured by a decrease in CRP levels, indicating that GP130 cannot be used as a biomarker for IL-6. Although the overall telopeptide profile for both NTx and CTx was variable following treatment, there was a hint of altered bone remodeling with an initial increase in both telopeptides after treatment, followed by an overall decrease below baseline levels in part 3. This observation, while consistent with lowered IL-6 bioactivity, is not robust enough to indicate either telopeptide as a biomarker of anti-IL-6 therapy.

The absence of an MTD further suggested dose escalation as a possibility for future development. In part 3, the dose regimen was intensified to 6 mg kg^–1^ q2w for all patients because the pharmacokinetic/pharmacodynamic model simulation determined it would be most effective in maintaining complete suppression of CRP (Puchalski *et al*, 2010). Although this regimen suppressed CRP in all patients, tumour response did not improve in part 3. The CRP data used for the modeling were not from a high sensitivity assay. If a high sensitivity CRP assay (i.e., LLOQ of 1 mg l^–1^) was used, a higher dose of siltuximab might have been required even in low-risk patients to decrease CRP to below the more sensitive LLOQ.

On the basis of the modified WHO criteria used in this study, the rate of CR or PR was low. However, considering the results of recent pivotal studies with other targeted therapies (Motzer and Bukowski, 2006; Escudier *et al*, 2007a) tumour response does not seem to be a prerequisite for clinical benefit. In reviewing selected computed tomography scans from one study site, increased tumour necrosis without significant tumour shrinkage was detected in two patients. This necrotic effect could have been because of an anti-angiogenic effect of siltuximab as previously reported (Zaki *et al*, 2004). Thus, other strategies for therapy with siltuximab, including combination with known active agents, can be considered. Additional measures of clinical efficacy (e.g., a delay in tumour progression) now shown to be relevant for non-cytotoxic agents should also be considered.

Siltuximab was very well tolerated in this study, particularly in comparison with available therapies. There were no dose- or schedule-dependent differences in the safety profile. A higher percentage of patients among the higher-risk patients enrolled in part 2 reported SAEs (24% with 3 mg kg^–1^ q3w, 40% with 6 mg kg^–1^ q3w,) than among the lower-risk patients enrolled in part 3 (15% with 6 mg kg^–1^ q2w). Only one SAE was considered possibly related to the study agent: cardiac failure in the 6 mg kg^–1^ q3w group. Five deaths occurred: two because of PD each in parts 1 and 2 and one because of cerebral haemorrhage secondary to metastatic disease in part 3. Additionally, no sample obtained from patients in any part of the study tested positive for immune response.

Despite the low single-agent activity seen in this study, the overall study results suggest the potential for further investigation of siltuximab either at higher doses and/or in combination with other active agents in the treatment of metastatic RCC.

## Figures and Tables

**Table 1:** Baseline demographics and disease characteristics

	**Part 1 Dose escalation**	**Part 2 Dose finding**			**Part 3 Dose confirmation**
**Dosage**	**1, 3, 6, or 12 mg kg^−1^ on days 1, 29, 43, 57**	**3 mg kg^−1^ q3w**	**6 mg kg^−1^ q3w**	**Total**	**6 mg kg^−1^ q2w**
Patients enrolled	11	18	20	38	20
Patients treated	11	17	20	37	20
Male	9 (82)	9/18 (50)	17 (85)	26 (68)	19 (95)
Caucasian	11 (100)	17/17 (100)	20 (100)	37 (100)	19 (95)
Age	60 (56, 73)	57 (39, 72)	57 (26, 82)	57 (26, 82)	62 (50, 77)
					
*Site of primary diagnosis*					
Right	4 (36)	8/17 (47)	12 (60)	20 (54)	13 (65)
Left	6 (55)	6/17 (35)	8 (40)	14 (38)	7 (35)
Bilateral	1 (9)	3/17 (18)	0	3 (8)	0
					
*Tumour histology*					
*N*	10	16	19	35	20
Clear cell	9 (90)	16 (100)	17 (90)	33 (94)	18 (90)
Papillary	0	0	1 (5)	1 (3)	1 (5)
Other	1 (10)	0	1 (5)	1 (3)	1 (5)
Patients with previous systemic cancer therapy	7 (64)	14/17 (82)	16 (80)	30 (81)	18 (90)
Patients with previous radiation therapy	4 (36)	1/17 (6)	8 (40)	9 (24)	1 (5)
Patients with RCC-related surgery	9 (82)	16/18 (89)	19 (95)	35 (92)	20 (100)

Abbreviation: RCC=renal cell carcinoma.

Data presented as *n* (%) or median (range).

**Table 2:** Summary of study-agent exposure and safety, treated patients

	**Part 1 Dose escalation**	**Part 2 Dose finding**			**Part 3 Dose confirmation**
**Dosage**	**1, 3, 6, or 12 mg kg^–1^ on days 1, 29, 43, 57**	**3 mg kg^–1^ q3w**	**6 mg kg^–1^ q3w**	**Total**	**6 mg kg^–1^ q2w**
Patients treated	11	17	20	37^a^	20
Patients treated with extended administrations	5 (45)	8 (47)	9 (45)	17 (46)	9 (45)
Doses received	4 (1, 8)	4 (2, 10)	4 (2, 8)	4 (2, 10)	6 (1, 13)
Days from first to final administration	57 (1, 64)	78 (29, 233)	64 (22, 212)	71 (22, 233)	71 (1, 239)
Patients who discontinued study agent	3 (27)	6 (35)	9 (45)	15 (41)	7 (35)
					
*Primary reason*					
Disease progression	1 (9)	5 (29)	8 (40)	13 (35)	4 (20)
Adverse event	1 (9)^b^	0	1 (5)^c^	1 (3)	1 (5)^d^
Other	1 (9)^e^	1 (6)^f^	0	1 (3)	2 (10)f,g
Patients who terminated study participation	3 (27)	4 (24)	4 (20)	8 (22)	3 (15)
					
*Primary reason*					
Disease progression	1 (9)	3 (17)	2 (10)	5 (14)	0
Death	0	0	2 (10)	2 (5)	1 (5)
Other	2 (18)e,f	1 (6)^f^	0	1 (3)	2 (10)^g^
Duration of follow-up, mean (days)	76	65	72	68	141
Patients with ⩾1 adverse events	11 (100)	17 (100)	19 (95)	36 (97)	19 (95)
Reasonably related	6 (55)	12 (71)	13 (65)	25 (68)	15 (75)
Grade 3 or higher	6 (55)	7 (41)	10 (50)	17 (46)	4 (20)
Patients with ⩾1 possible infusion reactions	1 (9)	2 (12)	4 (20)	6 (16)	3 (15)
Patients with ⩾1 serious adverse events^h^	5 (46)	4 (24)	8 (40)	12 (32)	3 (15)
Reasonably related	0	0	1 (5)	1 (3)	0
Death	2 (18)	0	2 (10)	2 (5)	1 (5)

a One patient assigned to 3 mg kg^–1^ did not meet entry criteria and consequently did not receive the study agent.

b Pneumonia.

c Cardiac failure.

d Proteinuria.

e Sponsor initiated.

f One patient withdrew consent.

g One patient with a history of renal failure and elevated hypertension had a recurrence of both.

h The following serious adverse events were reported: back pain, vomiting, arthralgia, peripheral motor neuropathy, anaemia, dyspnoea, pleural effusion, confusional state, general health deterioration, acute respiratory distress syndrome, bone pain, cardiac failure, acute pancreatitis, chest pain, sepsis, upper limb fracture, increased blood creatinine, cancer pain, spinal cord compression, vulval oedema, cerebral haemorrhage, upper abdominal pain, and leg ache.

Data presented as *n* (%) or median (range) unless specified otherwise.

**Table 3:** Summary of efficacy outcomes

	**Part 2 Dose finding**			**Part 3 Dose confirmation**
**Dosage**	**3 mg kg^−1^ q3w**	**6 mg kg^−1^ q3w**	**Total**	**6 mg kg^−1^ q2w**
Patients treated	17	20	37	20
Responders	11 (65)	10 (50)	21 (57)	13 (65)
Complete response	0	0	0	0
Partial response	1 (6)	0	1 (3)	0
Stable disease	10 (59)	10 (50)	20 (54)	13 (65)
Nonresponders	6 (35)	10 (50)	16 (43)	7 (35)
Progressive disease	6 (35)	10 (50)	16 (43)	7 (35)
Patients who received extended infusions	8 (47)	9 (45)	17 (46)	9 (45)
				
*Duration of overall response (CR* + *PR), days*				
*N*	1	0	1	0
Median	228	—	228	—
				
*Time to tumour progression (PD), days*				
Median (95% CI)	104 (60, 210)	66 (50, 189)	102 (52, 169)	80 (50, 130)
Observed	15 (88)	18 (90)	33 (89)	16 (80)
Censored	2 (12)	2 (10)	4 (11)	4 (20)

Abbreviations: CI=confidence interval; CR=complete response; PD=progressive disease; PR=partial response.

Data presented as *n* (%) unless specified otherwise.
